# A multidisciplinary approach to improve adherence to medical recommendations in older adults at hospital discharge: The APPROACH study protocol

**DOI:** 10.1371/journal.pone.0297238

**Published:** 2024-04-30

**Authors:** Bruno Micael Zanforlini, Sara Sambo, Maria Devita, Andrea Cignarella, Federico Vezzali, Silvia Sturani, Andrea Grandieri, Marianna Noale, Paola Siviero, Federica Limongi, Stefano Volpato, Giuseppe Sergi, Caterina Trevisan, the APPROACH working group

**Affiliations:** 1 Department of Medicine (DIMED), University of Padua, Padua, Italy; 2 Department of General Psychology (DPG), University of Padua, Padua, Italy; 3 Department of Medical Sciences, University of Ferrara, Ferrara, Italy; 4 Institute of Neuroscience – Aging Branch, National Research Council (CNR), Padua, Italy; 5 Aging Research Center, Karolinska Institutet, Stockholm, Sweden; Faculty of Pharmacy – Istinye University, TURKEY

## Abstract

**Introduction:**

Poor comprehension and medication adherence are common in older people, especially after hospitalizations, in case of changes or prescriptions of new therapeutic regimes. This randomized controlled trial aims to evaluate the effectiveness of an integrated approach in improving older individuals’ adherence to medical recommendations after hospital discharge.

**Methods:**

Data from an expected sample of 360 older inpatients (and their caregivers) will be collected. Medical recommendations’ understanding will be tested before and after the routine explanation received by in-charge physicians. Participants will be randomized in the control (usual care) and intervention group. The intervention consists of educational training by a multidisciplinary team (occupational therapist, dietician, and physician, in consultation with a pharmacologist) at hospital discharge and, after hospital discharge, receiving a phone recall checking for therapy adherence and having the possibility to contact the study team for potential related concerns. After 7 days, medication adherence will be assessed through structured phone interviews comparing ongoing and prescribed medications and by administering the 4-item validated Morisky, Green, Levine scale and the Medication adherence report scale (MARS-5). At 30 and 90 days from discharge, data on medication adherence, falls, rehospitalizations, and vital status will be collected through phone interviews and hospital records.

**Trial registration:**

**Registration:** NCT05719870 (clinicaltrial.gov). https://classic.clinicaltrials.gov/ct2/show/NCT05719870.

## Introduction

Noncommunicable diseases (NCDs) are the most frequent pathologies affecting the older population and are associated with high morbidity and disease burden [1]. In the last decades, clinical management of NCDs has made significant advancements. As a consequence, the prevalence of NCDs has increased, and older people are more and more likely to use multiple medications for the long-term management of NCDs and to have complex drug regimens [2]. In the real clinical world, this scenario may negatively affect medication adherence that, in older and multimorbid adults, has been estimated to be only around 50% [3,4]. According to the definition by the World Health Organization, medication adherence is “the extent to which a person’s behavior—taking medication, following a diet, and/or executing lifestyle changes, corresponds with agreed recommendations from a health care provider” [5]. Medication non-adherence can be intentional when the patient voluntarily decides not to follow medical recommendations or unintentional if it results from forgetfulness or not fully understanding medical instructions [6]. Medication non-adherence can also be classified based on the timing of medical prescriptions. In particular, primary medication non-adherence concerns newly prescribed treatments that have never been initiated, while secondary non-adherence includes behaviors leading to not correctly taking the medications when prescriptions are filled [7].

Medication adherence is a crucial issue after hospital discharge since changes in medication regimes or therapeutic requirements may represent new at-home challenges for patients and caregivers. Previous evidence showed that 56% of individuals aged 70 years or older reported discrepancies between the instructions received and their actual home medication just after 48 hours from discharge [8].

Scarce medication adherence represents a substantial risk factor for morbidity, mortality, and poor quality of life and is associated with high healthcare costs due to the lack of disease control and the need for hospitalizations [9–12]. There are different factors affecting medication adherence in the geriatric population: among patient-related factors, cognitive and psychological disturbances, poor health literacy, lack of medication knowledge, and misunderstanding of verbal instructions; among physician-related factors, poor communication; and, among system-based factors, lack of patient’s education [13]. An evidence-based review of randomized controlled studies found that behavioral/educational, pharmacist-led or reminder/simplification interventions can improve medication adherence in older adults [14]. Moreover, reports from different medical settings have shown that appropriate physician-patient communication on the diagnosis and treatment plan and shared decision-making could positively influence medication adherence and health-related outcomes [15].

To explore these issues in the older population, we designed a project to primarily test the effectiveness of a multidisciplinary intervention (vs. usual care) on adherence to medical recommendations in older inpatients after hospital discharge. Secondary aims were 1) to evaluate whether a multidisciplinary intervention could reduce the risk of incident falls, emergency department accesses, rehospitalizations, and mortality within the first three months after hospital discharge; 2) to assess the comprehension of medical recommendations of older patients (or their caregivers) at hospital discharge; and, 3) to identify the factors influencing poor comprehension and adherence to medical recommendations in older people.

## Materials and methods

### Study design and participants

In this randomized controlled clinical trial (RCT), participants will be recruited according to the following inclusion criteria:

- Patients (≥60 years old) admitted to the Geriatric Units of the University Hospitals of Padua (Italy) and Ferrara (Italy);
- Patients who will be discharged home;
- Patients for whom the expected survival is >7 days (considering that the study outcomes are assessed at 7, 30, and 90 days from hospital discharge and that patients with very short expected survival who are discharged at home usually are under palliative care, managed by a specialized team);
- Acquisition of informed consent to participate in the study.

Patients transferred to other medical wards or discharged to long-term care facilities will be excluded.

**Fig 1:**
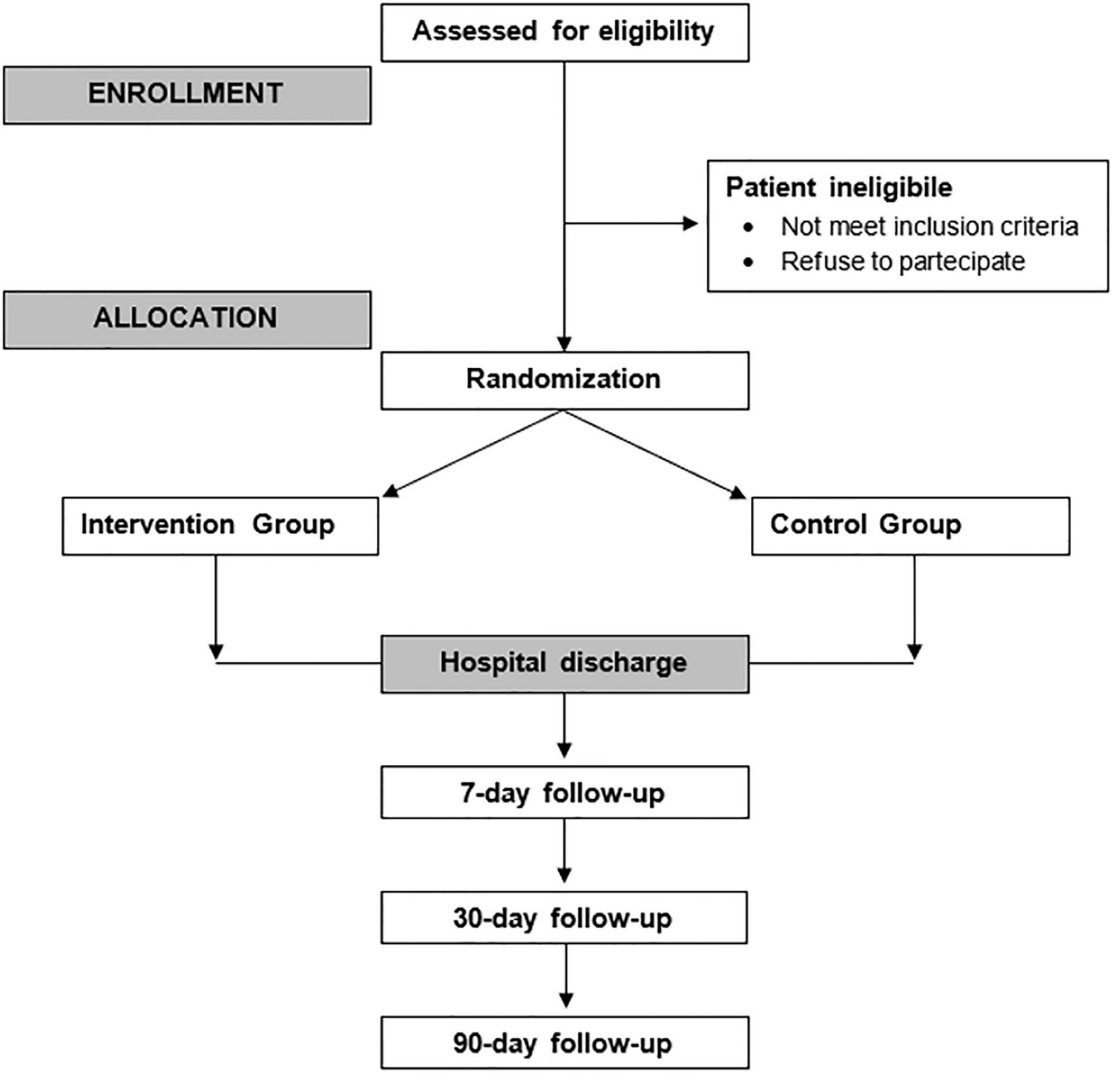
Flow-chart of the study.

For all those patients who are unable to manage therapy themselves at home, caregivers will be involved. Participants giving their consent will receive a detailed explanation of the project, its phases, and the purposes of the study. After hospital discharge, the total observation period for each study participant will be 90 days, as illustrated in Fig 1.

The APPROACH study protocol was approved by the Ethical Committee of the Padua Province for the coordinating center (Geriatric Unit, Department of Medicine, University of Padua, n. 5037/AO/21) and the Ethical Committee of the Area Vasta Emilia Centro della Regione Emilia-Romagna (CE-AVEC) for the collaborating center (Geriatric Unit, Department of Medical Sciences, University of Ferrara, n. 947/2021/Sper/AOUFe). All participants and, when applicable, their caregivers signed a written informed consent for participating in the study. The project complies with the principles of the Declaration of Helsinki.

### Study assessments

**Fig 2:**
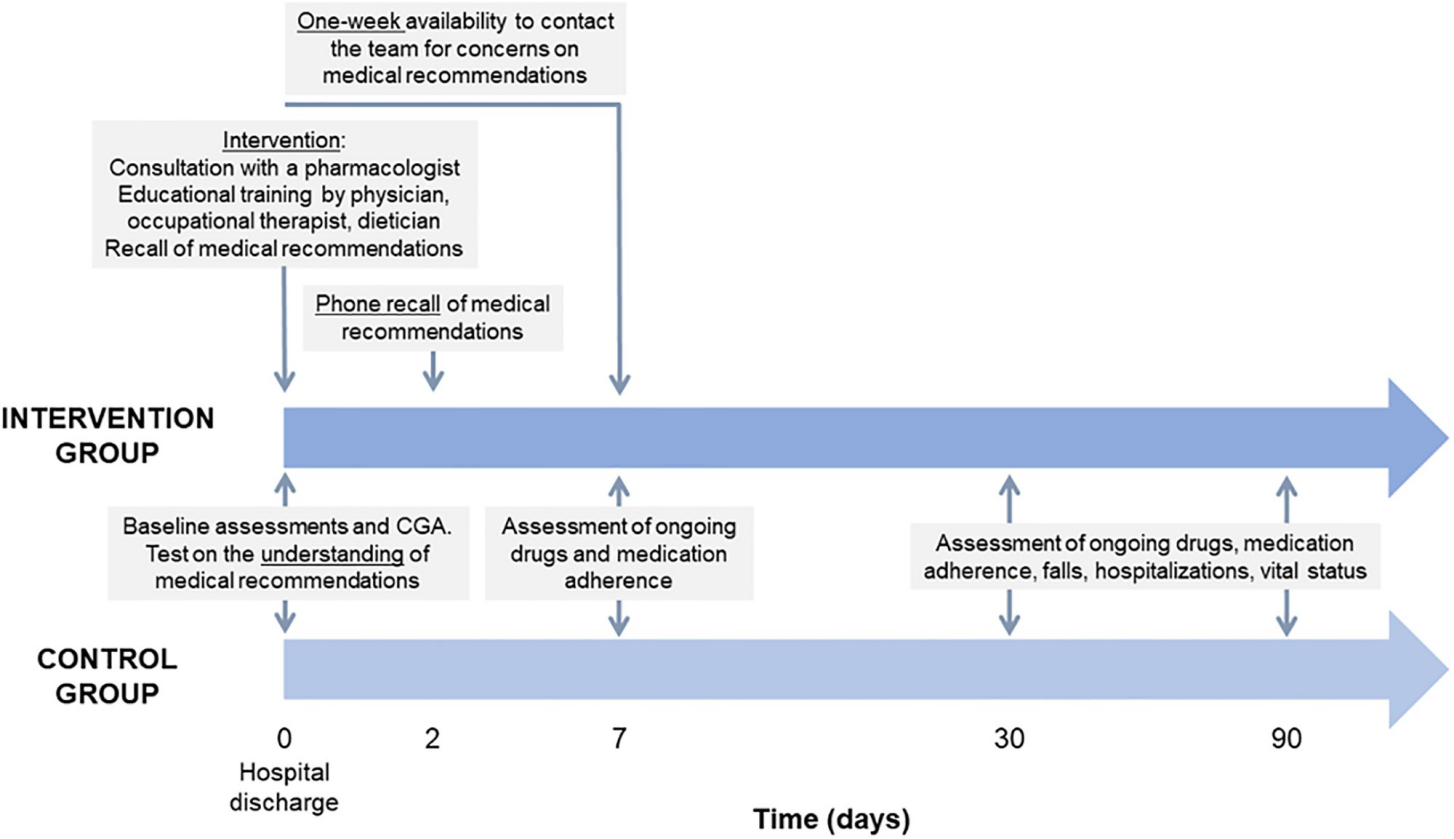
Timeline of the intervention and study assessments. Abbreviations: CGA, Comprehensive Geriatric Assessment.

Participants will undergo the following assessments (for a graphic summary, please see Fig 2):

Phase 1: data from the comprehensive geriatric assessment and additional information about the patient stay, prescribed therapies, health literacy, and sociodemographic characteristics of the caregiver (when applicable) will be collected at hospital discharge by trained researchers.
Phase 2: patients’ (or the caregivers, when applicable) understanding of medical recommendations before and after the explanation by the attending physician will be assessed at hospital discharge.
Phase 3: Participants will be randomly assigned to an intervention or control group.
Phase 4: After 7 days, adherence to medical recommendations at hospital discharge will be assessed through phone interviews with patients (or their caregivers, when applicable).
Phase 5: After 30 and 90 days, data on accidental falls, emergency department visits, rehospitalizations, and vital status will be collected both by accessing medical records and through phone interviews. During these interviews, the psychosocial impact of assistive technology will also be assessed.

### Randomization procedure

Participants will be randomly assigned to the multidisciplinary intervention group or the usual care procedure group in a 1:1 ratio, considering a computer simple randomization sequence generated by a statistician not having contact with the study participants. Outcome evaluators will be blinded to the participant’s allocation.

### Intervention and control

#### Intervention group

Before the beginning of the study, health professionals who administer the intervention (including 20 residents in Geriatrics and five geriatricians in the coordinating center, and 12 residents in Geriatrics, one nurse, and two geriatricians in the collaborating center) will be trained by the study coordinators to standardize the administration of interviews and scales for the patients’ assessments. The training will also include attending two seminars (in-person and video-recorded) led by the following specialists: a pharmacologist, with a focus on the burden and evaluation of inappropriate prescriptions and drug-drug interactions in older people with complex pharmacologic regimens; and a psychologist expert in communication, with a focus on the strategies to improving the skills for effective communication with patients and caregivers and to promoting a higher understanding of and adherence to medical recommendations. We decided to involve a pharmacologist considering that clinical pharmacists use the knowledge derived from pharmacology to achieve optimal therapeutic outcomes through the appropriate preparation and dispensing of medicines.

The three main components of the intervention administered to patients/caregivers are described below and include educational training, recall of the medical recommendations, and one-week phone availability to solve potential doubts about medical prescriptions.

First, at hospital discharge, patients will undergo educational training by 1) a physician (through an in-person meeting), to improve their knowledge of prescribed therapies’ purpose and use, correct dosing indications and potential interactions or drug-related problems; 2) a dietitian, to improve the adherence to nutritional recommendations (provided with written material); and 3) an occupational therapist (through an in-person meeting), to improve, sustain or restore the highest possible level of independence. For this step, a pharmacologist will be directly involved by physicians to provide counseling in case of any concerns on medication use (e.g. potential drug-drug interactions or inappropriate prescription) and for suggestions to improve medication adherence.

Secondly, 24 to 48 hours after hospital discharge, study participants will be contacted by phone to recall their therapies and to motivate them to adhere to medical recommendations.

Finally, during the first week after discharge, the intervention group will be offered the possibility to freely contact the study team by phone for 3 hours each day in case they have any concerns about prescribed treatments. Calls will be handled by a geriatrician who, based on the raised issue, will discuss the case with a pharmacologist, dietitian, or occupational therapist to address any concern appropriately, in addition to stressing the importance of medical compliance.

#### Control group

The control group will be discharged following standard care procedures and receive only appropriate corrections and clarifications if mistakes in comprehending the medical recommendations emerge in Phase 2.

### Data collection

Trained researchers at the Geriatric Units of the University Hospitals of Padua (Italy) and Ferrara (Italy) will collect data through the administration of structured interviews and standardized scales and questionnaires (for the complete list of the assessed variables, please see Table 1). For each scale or questionnaire, we will use the corresponding Italian version validated or used in previous studies.

**Table 1:** List of the domains evaluated and the used instruments.

Variable	Instrument/tool	Assessment Phase
**Sociodemographic and health information**	Age, sex, educational level, medical conditions	Hospital discharge
**Medications at hospital admission and discharge**	Information on the number and type of drugs used before the hospitalization and those prescribed at hospital discharge	Hospital discharge
**Multidimensional Geriatric Assessment**		
Nutritional status	Mini Nutritional Assessment—Short form (MNA-SF) [18] body weight and height (or knee height, for those with mobility limitations), self-reported body weight 6 months before the hospitalization, mid-arm circumference, waist circumference, and calf circumference	Hospital discharge
Functional status, physical performance and frailty	Activity of Daily Living (ADL) [20], Instrumental ADL (IADL) [21], Exton-Smith Scale (ESS) [19] Handgrip, SARC-F [23] FRAIL questionnaire [22]	Hospital discharge
Cognitive status	Short Portable Mental Status Questionnaire (SPMSQ) [17]	Hospital discharge
Burden of disease	Cumulative Illness Rating Scale (CIRS) [16]	Hospital discharge
Global prognosis	Multidimensional Prognostic Index (MPI)	Hospital discharge
**Hospital stay duration and discharge diagnosis**	Length of hospital stay (days from hospital admission and discharge) Discharge diagnosis coded by the International Classification of Diseases—10^th^ revision	Hospital discharge
**Self-assessment of understanding of medical instructions**	Brief health literacy questionnaire [26]	Hospital discharge
**Comprehension of medical recommendation**	Written ad hoc questionnaire developed by the researchers regarding number and type (drug name, dose and frequency or other recommendations) of mistakes in recalling medial recommendations	Hospital discharge
**Adherence to medical recommendations**	4-item validated Morisky, Green, Levine scale [24]. Medication adherence report scale (MARS-5) [25]	Hospital discharge, 7-, 30- and 90-day follow-up
**Variations of therapy and its motivation**	Ad hoc questionnaire administered by phone interviews	Hospital discharge, 7-, 30- and 90-day follow-up
**Number of falls, emergency department visits, and rehospitalizations after hospital discharge**	Ad hoc questionnaire administered by phone interviews	Hospital discharge, 7-, 30- and 90-day follow-up
**Vital status**	Phone interviews and hospital records	7-, 30- and 90-day follow-up
**Psychosocial Impact of Assistive Devices**	PIADS Instrument (Psychosocial Impact of Assistive Devices Scale) [27]	30- and 90-day follow-up

#### Participants’ characteristics

At hospital discharge (Phase 1), participants will be assessed about sociodemographic data (age, sex, educational level, previous work, marital status, number of living children, living arrangement) and current (cause and duration of the hospitalization) and anamnestic clinical status (Cumulative Illness Rating Scale [16] [CIRS], number and type of medications used before the hospital admission). Moreover, data from the routine multidimensional geriatric assessment will be recorded, including the evaluation of cognitive function (Short Portable Mental Status Questionnaire [17] [SPMSQ]), nutritional status (Mini Nutritional Assessment—Short form [18] [MNA-SF] and anthropometric data, such as body weight and height, waist, mid-arm, and calf circumference), risk of developing pressure ulcers (Exton-Smith Scale [19] [ESS]) and functional ability (Activity of Daily Living (ADL) [20] and Instrumental ADL [21] [IADL]). In addition, participants will be evaluated concerning frailty (FRAIL questionnaire [22]), sarcopenia (SARC-F questionnaire, handigrip [23]), medication adherence before the hospitalization (4-item validated Morisky, Green, Levine scale [24], and Medication adherence report scale [25] [MARS-5]), health literacy [26] (Brief health literacy questionnaire), and the psychosocial impact of assistive technology on participants who will be using any device (Psychosocial Impact of Assistive Devices Scale [27] [PIADS]).

#### Primary and secondary outcomes

At hospital discharge (Phase 2), researchers will interview the patients (or their caregivers, when applicable) about the comprehension of the medical recommendations reported in the discharge summary. In particular, before and after the explanation of the medical recommendations by the physician, possible mistakes in the understanding of prescriptions will be recorded and classified based on the fact they concern the identification (e.g. do not spell the drug correctly or do not know the role of the medication), dosage, number of administrations, and time of administration of each drug.

After 7, 30, and 90 days from hospital discharge (Phase 4 and 5), patients will undergo a phone interview lasting approximately 30 minutes to obtain information on the ongoing treatments (recording name, dose, and administration time of the therapies taken). In particular, for each medical recommendation included in the discharge summary, we will assess whether the patient follows that prescription by taking the correct drug at the correct dosage and administration time. From the ratio between the number of medications taken with full adherence and the total number of prescriptions included in the discharge summary, we will derive the proportion to which the patient adheres to the given medical recommendations (primary outcome). As an additional measure of medication adherence, we will consider the results at the 4-item validated Morisky, Green, Levine scale [24] and MARS-5 [25].

Moreover, data on incident falls, hospitalizations and vital status will be recorded from phone interviews and medical records at the local hospitals. These outcomes were selected in light of the current literature supporting the impact of poor medication adherence on the risk of accidental falls and the need for medical attention [26,27]. At the 30- and 90-day follow-up, the psychosocial impact of assistive devices will be evaluated with the PIADS instrument.

### Statistical analysis

#### Sample size

The study sample size was estimated by using PASS 2008 (version 08.0.16). The difference in the level of adherence to medical recommendations of patients undergoing the multidisciplinary intervention vs. usual care after 7 days since hospital discharge was considered as the primary outcome. A previous study involving adults discharged from the emergency department found a correct recall percentage of 64.6% among patients assisted with standard care and 79.4% among those who underwent an educational/reminder intervention through the teach-back method [28]. Considering a power of 80%, an alpha of 0.05, and a drop-out rate of 20%, a total of 180 patients per group will be required for our study, with a sample size of 360 patients.

#### Planned statistical analyses

Baseline characteristics of study participants will be expressed as mean ± standard deviation (for normally distributed quantitative variables), median and interquartile range (IQR, for non-normally distributed quantitative variables), and frequencies and percentages for qualitative variables. The normal distribution of the quantitative variables will be tested by the Shapiro-Wilk test and graphical evaluation. Comparison of patients’ (or their caregivers’) understanding of medical recommendations before and after explanation by treating physicians will be performed using Student’s t-test for paired samples (or Wilcoxon’s test for quantitative variables not normally distributed). As a measure of understanding, we will consider the total number of errors and the number of specific mistakes about the identification, dosage, number of administrations, and time of administration of each prescription. As adherence measures, we will consider the proportion of prescriptions taken with full adherence and the score at the 4-item validated Morisky, Green, Levine scale [24] and MARS-5 [25]. Factors potentially associated with poor understanding and adherence to medical recommendations will be identified by logistic regression analysis, and the strength of these associations in unadjusted and multi-adjusted models will be expressed as odds ratios with 95% confidence intervals (95%CI). The impact of medication adherence and the proposed intervention on incident falls, rehospitalizations, and mortality will be assessed through Cox regression analyses and expressed as hazard ratios and 95%CI. Potential interactions between baseline factors influencing study outcomes will be explored by including the multiplicative interaction terms in the model. In this regard, special attention will be paid to investigating possible sex differences or disparities due to socioeconomic level or the type of caregiver in the associations tested.

Analyses will be performed using statistical software SPSS and R. All statistical tests will be two-tailed, and results will be considered statistically significant with a p-value <0.05.

## Discussion

This project aims to underline the importance of a comprehensive evaluation and communication with older people that may help clinicians plan personalized interventions, better control chronic conditions, and support capacity-enhancing behaviors [29]. Overall, this should facilitate the recovery of older adults after hospital discharge by improving their medication adherence and functional abilities at home.

It is common for older patients at hospital discharge to be unable to recall their diagnoses and the names, roles, and adverse effects of the prescribed medications [14,15]. In particular, written discharge instructions alone have emerged as poorly understood by approximately 45% of patients [17]. To address this issue and in light of the gaps arising from some reviews on this topic [14,15], we have proposed a project that may be novel and relevant for clinical practice.

First, different from previous studies that focused on the effectiveness of a single domain investigation (behavioural/educational, pharmacist-led, reminder/simplification [14]), the project will test the potential benefits of a multidimensional approach in improving medication adherence. This approach is more likely to identify the multiple factors influencing patients’ adherence to medical recommendations, including not only sociodemographic and health characteristics but also cognitive or functional deficits [13]. As suggested by a recent consensus, tackling the complexity of non-adherence would require a multi-stakeholder, patient-centered approach that should involve different players and services [15]. Training health professionals on communication skills will facilitate achieving the goals in these first steps and may increase physician and patient satisfaction, reduce burnout, and support therapeutic alliance [30,31]. Moreover, this multidisciplinary intervention of the study participants may positively influence patients’ in different health domains and allow us to identify factors that, interacting with medication adherence, could influence health-related outcomes in such individuals. Indeed, by improving adherence to medical recommendations, we expect to find also a lower risk of adverse health-related outcomes (i.e., falls, hospitalizations, and mortality).

Second, in addition to its multidisciplinary nature, the proposed intervention is characterized by low costs, low technologic requirements −which, to date, may still be a factor that hampers the adherence of patients in the oldest classes of age in Italy− and easy implementation. These features strengthen the feasibility of this intervention in a real-life setting.

Third, another strength of this project is the use of different methods to comprehensively capture the level of the patient’s adherence to medical instructions at hospital discharge since studies on the topic have shown a wide heterogeneity in this assessment. As suggested by current guidelines [32], patients’ adherence will be estimated using multiple assessment methods based on structured face-to-face interviews and validated scales.

On the other hand, among the possible obstacles to the project implementation are difficulties in participants’ enrolment and attrition. In particular, the enrolment of the study participants and the project’s activities could be slowed down by the COVID-19 pandemic, whose related restrictions could hamper the interactions between different health professionals and the communication with patients’ caregivers. Moreover, randomization is not performed by the enrollment site, and this cannot completely rule out that part of the trained team may explain the medical recommendations to the control group more extensively than usual during the routine discharge meeting. This issue could eventually lead to an underestimation of the effectiveness of the intervention. However, the components of further explanation and recall of the medical recommendations solely characterize the intervention and represent a decisive addition to the routine practice that could positively influence patients’ adherence.

Overall, the study will benefit our primary audience, namely the patients involved, and may also offer helpful information and support to caregivers of clinically complex individuals. It could also provide useful insights for implementing this integrated care model in different settings. For instance, a possible project extension will be integrating this multidisciplinary intervention at hospital discharge with a closer involvement and collaboration of general practitioners. In that way, patients’ functional recovery after hospital discharge will be further promoted by strengthening the continuum between secondary and primary care.

As regards the dissemination activities, the results of this project will lead to the production of scientific papers that will be submitted for publication in international biomedical journals. The participation of study team members in national and international conferences will offer a further opportunity to disseminate the results of our project and their potential implications. Physicians working at the local hospital will be updated over the study period on the ongoing project and, after its end, will be invited to a dedicated seminar to show the study results and to increase awareness of the importance of adequate communication and education of the patient at hospital discharge by experts in communication and skilled pharmacologists. Moreover, the team will organize educational events to present the study findings and stress the importance of full adherence to medical recommendations to promote healthy ageing.

## Conclusions

In conclusion, the APPROACH trial will test the effectiveness of a multidomain intervention with an integrated care approach to improve the comprehension and adherence to medical recommendations of older people at hospital discharge, compared with usual care. This intervention is expected to facilitate the returning home and functional recovery of older patients after hospital discharge and influence other health-related outcomes, reducing the risk of accidental falls, hospital readmissions, and mortality.

## Supporting information

S1 File Supplementary material. Complete list of the APPROACH working group (in alphabetic order).
(DOCX)

S2 File Spirit checklist. Scientific, ethical, and administrative recommendations for the drafting of clinical trial protocols.
(DOC)
