# Supplementary material for: A multidisciplinary approach to improve adherence to medical recommendations in older adults at hospital discharge: The APPROACH study protocol
Source: PLoS One. 2024 Apr 30;19(4):e0297238. doi: 10.1371/journal.pone.0297238 (PMC11060519; doi:10.1371/journal.pone.0297238)
Supplement: S1 File — Complete list of the APPROACH working group (in alphabetic order). (DOCX) [file pone.0297238.s001.docx]

**SUPPLEMENTARY MATERIAL**

**Appendix 1. Complete list of the APPROACH working group (in alphabetic order)**

Viviana Bagalà (University Hospital of Ferrara, Ferrara, Italy), Simone Balanzoni (University Hospital of Ferrara, Ferrara, Italy), Elena Barbieri (University Hospital of Ferrara, Ferrara, Italy), Valentina Bernardi (University of Padua, Padua, Italy), Anna Bertocco (University of Padua, Padua, Italy), Francesca Bizzotto (University of Padua, Padua, Italy), Marianna Boccafogli (University Hospital of Ferrara, Ferrara, Italy), Carlotta Bortoluzzi (University of Padua, Padua, Italy), Alessandra Cannata (University of Ferrara, Ferrara, Italy), Fabio Celi (University of Padua, Padua, Italy), Francesca Cera (University of Padua, Padua, Italy), Andrea Cignarella (University of Padua, Padua, Italy), Chiara Curreri (University Hospital of Padua, Padua, Italy), Francesca Curri (University of Padua, Padua, Italy), Laura Dal Gesso (University of Padua, Padua, Italy), Marina De Rui (University Hospital of Padua, Padua, Italy), Maria Devita (University of Padua, Padua, Italy), Benedetta Di Marzio (University of Padua, Padua, Italy), Nicolò Gentili (University of Padua, Padua, Italy), Andrea Grandieri (University Hospital of Ferrara, Ferrara, Italy), Alessandro Lazzarin (University of Padua, Padua, Italy), Maria Leonardo (University Hospital of Ferrara, Ferrara, Italy), Federica Limongi (Institute of Neuroscience, National Research Council, Padua, Italy), Serena Lorenzo (University of Padua, Padua, Italy), Lisa Marzano (University Hospital of Ferrara, Ferrara, Italy), Eleonora Mizzon (University of Padua, Padua, Italy), Marianna Noale (Institute of Neuroscience, National Research Council, Padua, Italy), Giulia Pampolini (University Hospital of Ferrara, Ferrara, Italy), Federica Piccione (University of Padua, Padua, Italy), Nicola Rampon (University of Padua, Padua, Italy ), Francesca Remelli (University of Ferrara, Ferrara, Italy), Alberto Rossi (University of Padua, Padua, Italy), Arianna Sala (University Hospital of Ferrara), Sara Sambo (University of Padua, Padua, Italy), Claudia Sartori (University of Padua, Padua, Italy), Giuseppe Sergi (University of Padua, Padua, Italy), Paola Siviero (Institute of Neuroscience, National Research Council, Padua, Italy), Silvia Sturani (University of Padua, Padua, Italy), Sara Tamascelli (University of Ferrara, Ferrara, Italy), Giulia Tasso (University of Padua, Padua, Italy), Marta Tinazzo (University of Padua, Padua, Italy), Caterina Trevisan (University of Ferrara, Ferrara, Italy; University of Padua, Padua, Italy), Jessica Vanin (University of Padua), Federico Vezzali (University Hospital of Ferrara, Ferrara, Italy), Stefano Volpato (University of Ferrara, Ferrara, Italy), Bruno Micael Zanforlini (University Hospital of Padua, Padua, Italy).
